# An open-source low-power wheel detector node for urban rail monitoring

**DOI:** 10.1016/j.ohx.2026.e00830

**Published:** 2026-08-21

**Authors:** Eren Erdi, Emrah Sarioglu, Baris Oguz Gurses, Aysun Baltaci

**Affiliations:** a Department of Mechanical Engineering, Graduate School of Natural and Applied Sciences, Ege University, Bornova, Izmir 35100, Turkey; b 3rd Regional Directorate, Turkish State Railways (TCDD), Izmir 35220, Turkey; c Department of Mechanical Engineering, Ege University, Bornova, Izmir 35100, Turkey

**Keywords:** Open-source hardware, Wheel-passage monitoring, Low-power electronics, Contact mechanics, Electromechanical system

## Abstract

Wheel-passage monitoring is relevant to non-safety–critical railway applications such as temporary traffic observation, maintenance support measurements, and open-hardware field experimentation. This study presents an open-source, low-power wheel detector developed as a monitoring-oriented railway sensing node for operation under real field conditions. The proposed hardware combines a mechanically preloaded pedal, a reed-switch-based triggering arrangement, and an ultra-low-power electronic subsystem built around a prototype control platform. In deep-sleep mode, the prototype consumes approximately 10 µA. The mechanical behavior of the detector was analysed using MATLAB/Simscape, focusing on wheel–pedal contact, preload selection, transient displacement response, and contact-pressure evolution with wheel speed. Preload optimization based on an overshoot criterion showed that 24 mm is the minimum preload required to eliminate secondary penetration. At 90 km/h, the model predicted full damping within 17.076 ms, remaining below the 30 ms digital filtering window and thereby supporting stable single-event generation. A damping sensitivity analysis showed that this timing margin is preserved under reasonable variation of the assumed structural damping. Within the adopted reduced-order 2D plane-strain model with isotropic hardening, the cyclic elastoplastic assessment predicted a shakedown-like response, with residual vertical indentation approaching 0.70 mm, well below the 5 mm triggering allowance of the reed-switch mechanism. A field-installed prototype was evaluated over the 0–90 km/h speed range. A total of 500 monitored wheel-passage events were assessed, with no missed detections and no visible wheel-flange deformation after testing. The results support the feasibility of a low-cost, ultra-low-power, open-source wheel-passage detector for non-safety–critical railway monitoring.

## Graphical abstract

**Figure:**
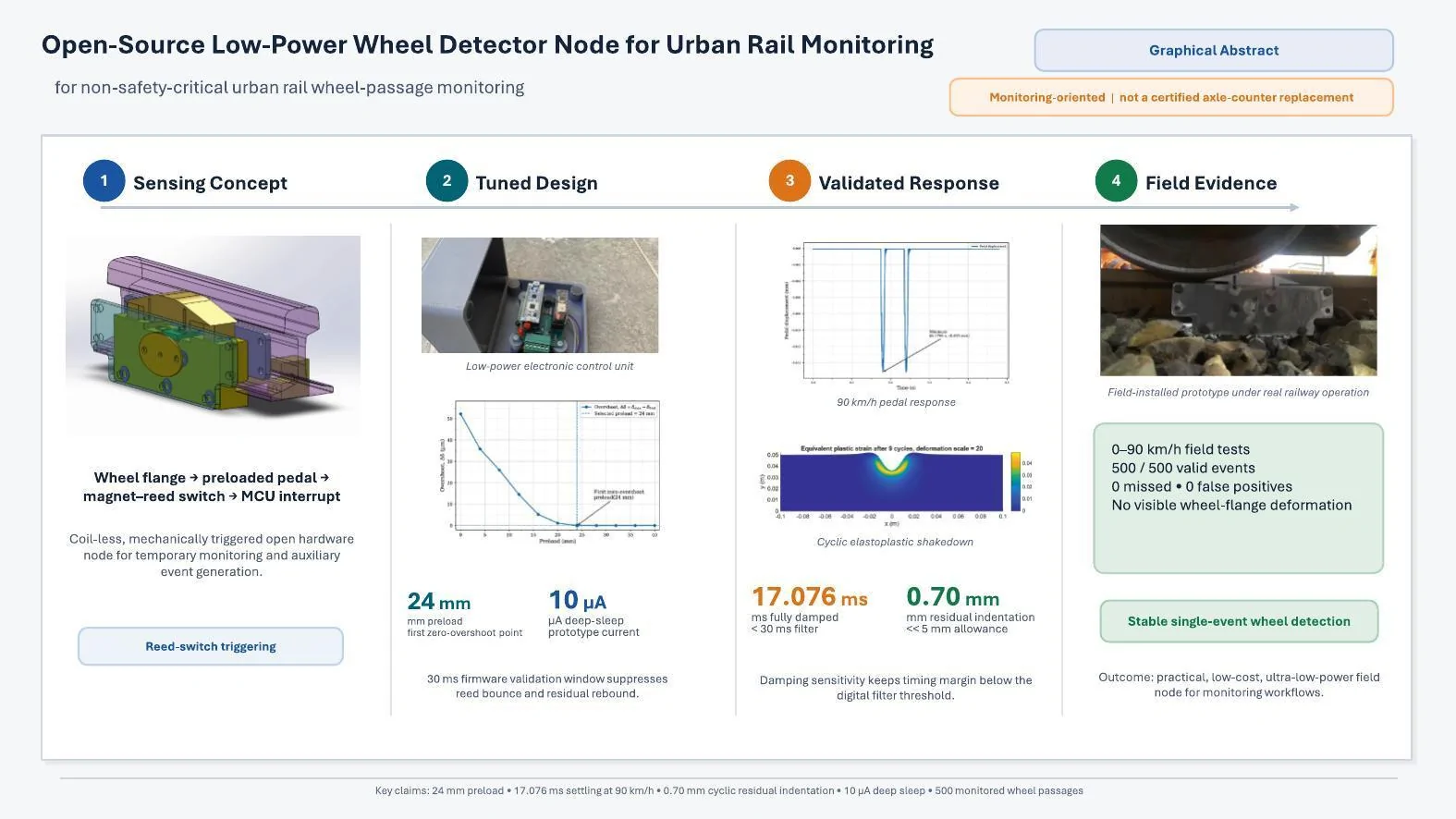

Specification table

**Table:** 

Hardware name	Open-source pedal-based railway wheel detector
Subject area	• Engineering and materials science • Civil and transportation infrastructure monitoring • Open-source alternatives to existing railway wayside detectors
Hardware type	• Measuring physical properties and field sensors • Existing infrastructure modification / trackside sensing hardware
Open-source license	CC BY 4.0
Cost of hardware	approx. 500 €
Source file repository	https://data.mendeley.com/datasets/zc249jgz2x/2

## 1 Hardware in context

Railway infrastructure increasingly relies on sensor-based condition monitoring to support maintenance planning, asset inspection, and data-driven decision making. Recent reviews show that modern railway monitoring is progressively moving toward digitalized and predictive maintenance frameworks in which sensors installed on trackside assets and vehicles provide real-time information on vibration, temperature, geometry, and other condition indicators. IoT-enabled sensing, distributed data acquisition, and low-power field nodes are becoming increasingly relevant in this transition toward smart railway maintenance [1], [2].

Within this broader landscape, wayside monitoring systems already play an established role in railway practice. Review articles on wayside train monitoring and wayside condition monitoring describe a wide range of trackside systems used for safety- and maintenance-related observation of rolling stock and infrastructure. These include devices for wheel, axle, bearing, and impact monitoring, as well as broader condition-monitoring architectures intended to support diagnosis and maintenance planning. At the same time, the literature also highlights that existing wayside solutions are often associated with permanent installation, proprietary architectures, higher deployment complexity, or application-specific infrastructure requirements [3], [4], [5].

In parallel, distributed sensor-network approaches have also been explored in railways for condition monitoring of infrastructure, track equipment, bogies, wheels, wagons, and other assets. Surveyed work on wireless railway sensor networks shows that such systems are attractive because they reduce manual inspection effort, support automated event detection, and enable scalable monitoring of geographically distributed assets. This creates a practical niche for compact, low-power, field-deployable sensor nodes that do not aim to replace certified train-detection or signalling subsystems, but instead support maintenance-oriented monitoring, temporary measurements, and experimental data acquisition [6].

The present work addresses this niche with an open-source, low-power wheel detector intended for monitoring-oriented railway use rather than direct integration into certified signalling functions. The proposed device is not presented as a replacement for track circuits, axle counters, or other safety–critical train-detection systems. Instead, it is conceived as a compact, battery-operated wheel-passage monitoring node that can be installed with limited infrastructure, minimal trackside intervention, and straightforward adaptation to project-specific monitoring tasks. In this role, the detector can support temporary wheel-passage logging during maintenance campaigns, proof-of-concept evaluation of rail-side sensing concepts, trigger generation for auxiliary data-acquisition systems, local event logging at locations without fixed power supply, and distributed IoT-style monitoring architectures in which wheel-passage events are used as edge-level inputs for higher-level maintenance analytics. The open-hardware implementation further supports redesign, replication, and low-cost experimentation in non-safety–critical railway applications [4], [6].

In addition, urban rail systems encompass a broad range of guided public-transport modes including metros, trams, and light rail, as reflected in European standardization guidance [7]. Across these modes, published rolling-stock and system studies commonly report maximum operating speeds up to about 80 km/h for light-rail and metro vehicles [8], [9]. In this work, we scope the proposed wheel detector node and its subsequent mechanical characterization to urban rail operating conditions up to 90 km/h, representing a conservative upper-bound regime within the intended deployment scope.

## 2 Hardware description

The proposed wheel detector node comprises a mechanical sensing assembly and an electronic/firmware subsystem. Wheel passages are converted into discrete detection events using a coil-less, mechanically triggered reed switch mechanism integrated into a spring-loaded pedal guided within a rail-mounted carrier. Electronics implement ultra-low-power, interrupt-driven event acquisition to support battery-operated deployments and firmware-level event validation.

### 2.1 Mechanical subsystem

**Fig. 1:**
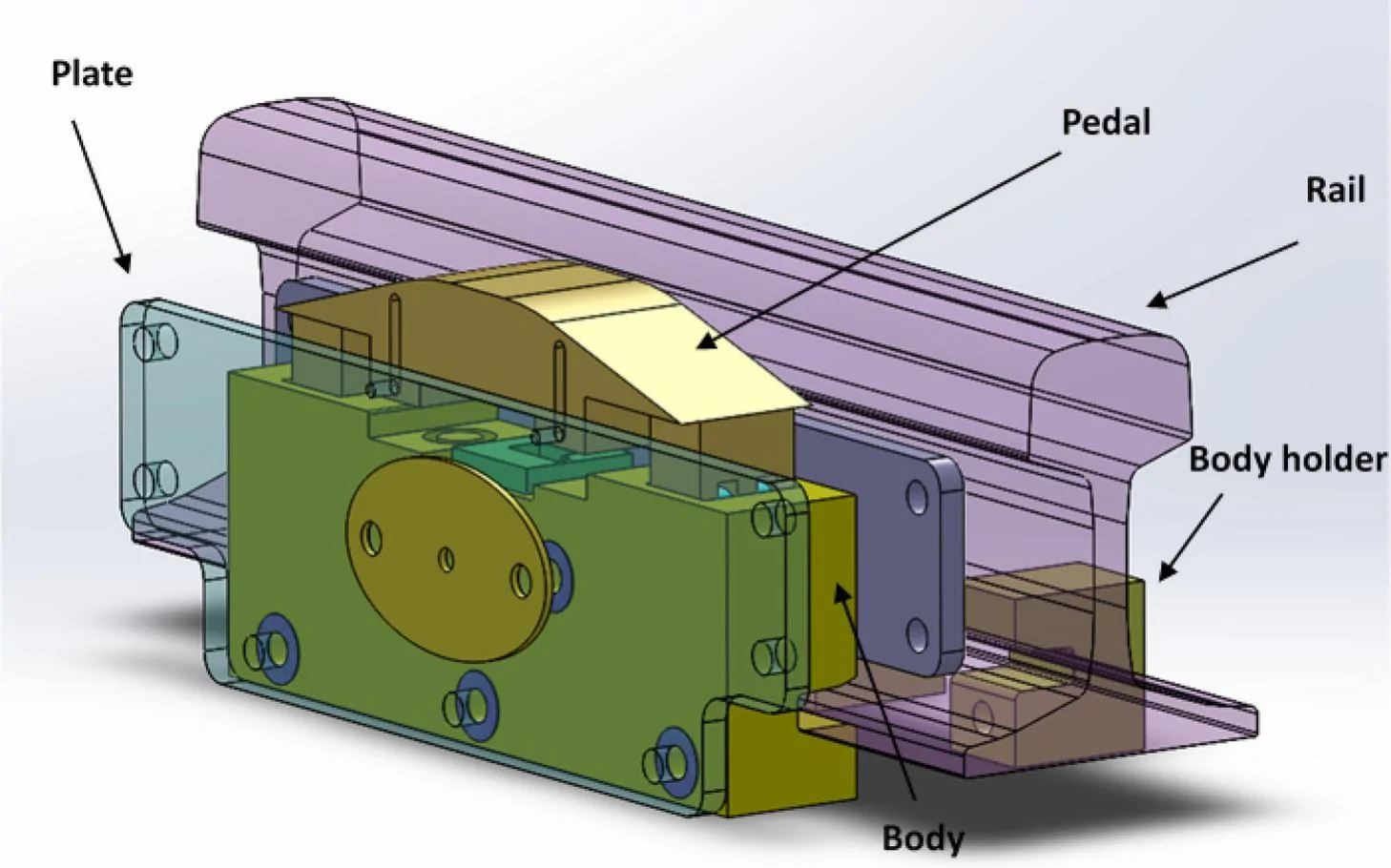
Wheel detector CAD model.

The proposed wheel detector unit is a mechanical assembly mounted on the rail and consists of a housing, pedal, and spring, as illustrated in the CAD model shown in Fig. 1. The wheel-pass-induced vertical displacement of the pedal is converted into an electrical trigger by a reed switch located inside the housing. The reed-switch arrangement comprises a permanent magnet and a reed sensor; in this design, the magnet is embedded in the pedal whereas the reed sensor is placed inside the housing, such that the magnet–sensor gap decreases during pedal motion and the switch is triggered. The selected reed sensor is IP67-rated, with a nominal sensing distance of 13 mm and a switching capability of 500 Hz. Based on the minimum bogie wheelbase values specified in UIC Code 511 (≈1.5–2.0 m), the maximum wheel-passage event frequency at 90 km/h (25.0 m/s) is on the order of f_max_ = v / L ≈ 25.0/2.0 to 25.0/1.5 ≈ 12.5 to 16.67 Hz, i.e., orders of magnitude below 500 Hz [10], [11]. This switching capability provides a sufficiently fast dynamic response for reliable event generation under repeated triggering conditions. An IP67 rating denotes complete protection against dust ingress and resistance to temporary water immersion (up to 1 m for 30 min) as defined by the IP code standard [12].

Mechanical contact between the railway vehicle and the wheel detector occurs at the interface between the wheel flange and the pedal. As the flange loads the pedal, the pedal is displaced downward along a guided vertical path, reducing the magnet–reed gap until the magnet enters the sensing range of the reed switch and an electrical trigger is generated. After the wheel passage, the contact is released and the spring restores the pedal to its initial position, resetting the detector for the next event.

The wheel flange heights vary across the railway rolling stock. According to international railway standards, the flange height depends on the wheel diameter and typically ranges from 28 mm to 36 mm. In urban rail systems, the flange height of powered and towed vehicles is commonly standardized to 28 mm [13]. Accordingly, the wheel node was installed such that the pedal surface was positioned 14 mm below the top of the rail head, and the geometry was designed to ensure reliable detection of wheels with a 28 mm flange height. This mounting offset was selected to ensure that wheel–pedal contact forms reliably despite manufacturing/wear tolerances in flange height, and to keep the contact region as much as possible within the flange area. Key mechanical and triggering parameters are summarized in Table 1.

**Table 1:** Mechanical and triggering parameters.

**Parameter**	**Symbol**	**Value**	**Unit**	**Notes**
Housing / carrier material	–	Al 6063-T6	–	Main mechanical structure
Pedal top offset from rail head	Δh	14	mm	Pedal surface positioned below rail head to ensure flange–pedal contact
Maximum pedal stroke	S_max_	14	mm	Vertical travel under wheel passage
Spring preload (compression)	x_0_	24	mm	Preloaded at assembly
Magnet–reed gap (max.)	h_mr_	27	mm	Adjustable between 22–27 mm
Reed sensing distance	L_r_	13	mm	From reed sensor specification [11]
Reed switching capability	f_sw_	500	Hz	From reed sensor specification [11]

The reed sensor is mounted at the base of the housing, oriented toward the permanent magnet embedded near the upper surface of the pedal. The design allows for an adjustable clearance of up to 5 mm in the vertical axis for sensor placement in the reed sensor housing depth. The pedal’s vertical motion under mechanical contact is provided by a helical compression spring, while lateral forces are carried by bearings located at side constraints of the pedal. The spring is installed with preload, intended to limit post-contact oscillations of the pedal, accelerate settling back to the equilibrium position, improve stability against vibration, and absorb peak shocks at the initial contact event to achieve a more controlled compression response. Spring design parameters used in the mechanical subsystem are summarized in Table 2 [14], [15].

**Table 2:** Key spring design parameters.

**Parameter**	**Symbol**	**Value**	**Unit**	**Notes**
Spring end type	−	Plain open end	–	[14], [15]
Number of springs	n	2	–	Two identical springs
Spring wire diameter	d	4.5	mm	[14], [15]
Active coil count	N_e_	5	–	[14], [15]
Mean coil diameter	D_m_	21	mm	[14], [15]
Spring index	C	4.67	–	[14], [15]
Shear modulus	G	80	GPa	[14], [15]
Spring constant (per spring)	k	88.56	kN/m	[14], [15]
Free length	L_0_	64.5	mm	[14], [15]

The wheel–pedal contact dynamics and the resulting pedal response are subsequently analysed in MATLAB/Simscape to characterize the event-generation envelope under the operating conditions considered in this study.

### 2.2 Electronic and embedded software subsystem

The electronic and embedded software subsystem converts the reed-switch transition into a robust, time-stamped detection event while maintaining ultra-low-power operation for battery deployment. The front-end conditions the reed input to suppress contact bounce and provide a stable logic-level transition to the microcontroller, and the microcontroller implements interrupt-driven wake-up, event validation, and counting logic. An optional communication interface can be integrated in future revisions without altering the sensing principle.

**Fig. 2:**
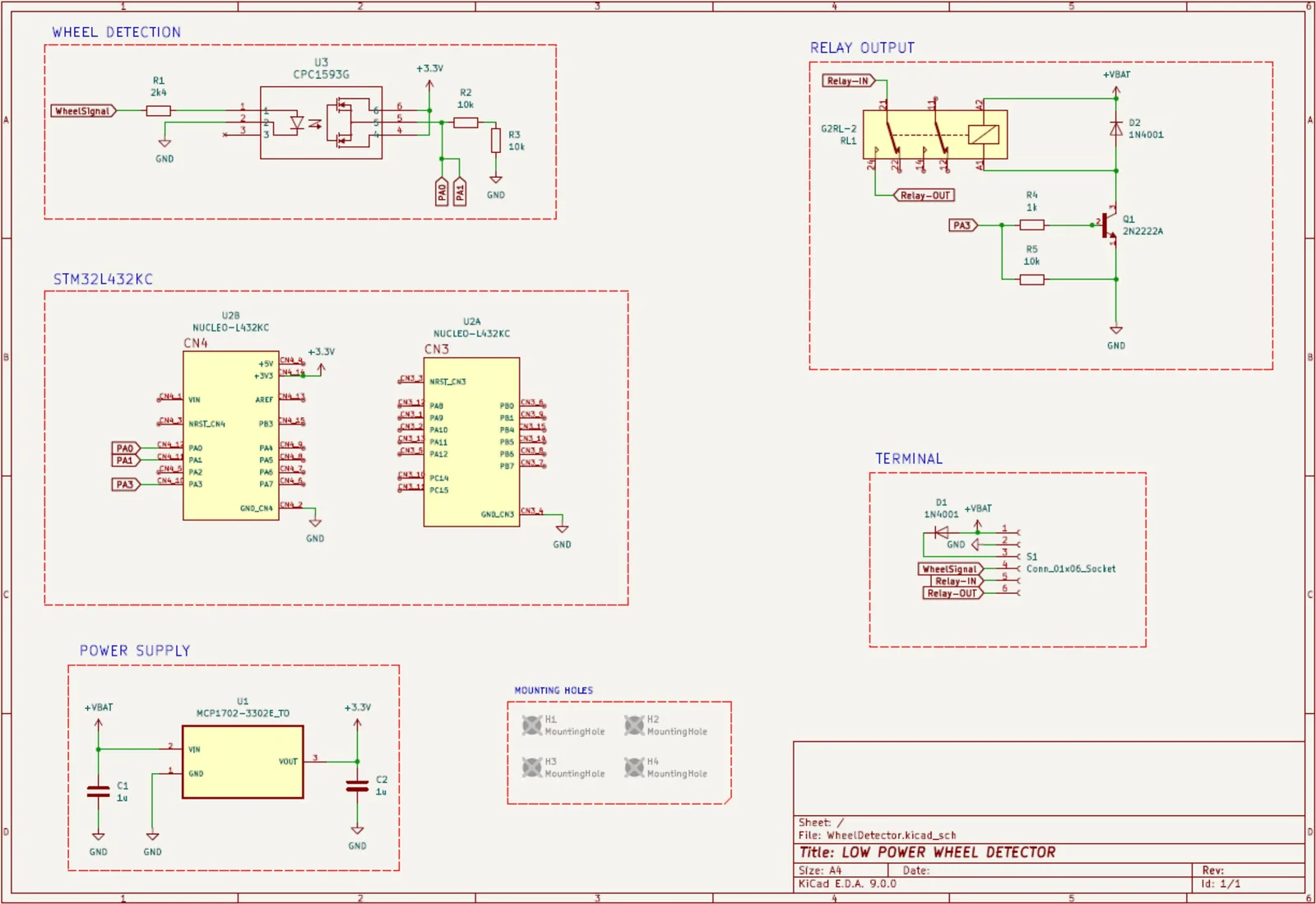
KiCad schematic.

In the electronic subsystem, an STM32L432KC-based microcontroller was selected to achieve ultra-low-power operation while maintaining access to a mature and widely supported development ecosystem. For voltage regulation, a low-noise, low-quiescent-current LDO was preferred over a switching regulator in order to minimize both standby power consumption and electrical noise. The electronics were designed to operate over an input range of 3.7–13 VDC. The deep-sleep current consumption of the prototype, including the regulator and input stage, was measured as approximately 10 µA using a Unit-T UT98X digital multimeter. A simplified KiCad schematic highlighting the power-regulation path and the reed-input conditioning/interrupt interface is presented in Fig. 2.

**Fig. 3:**
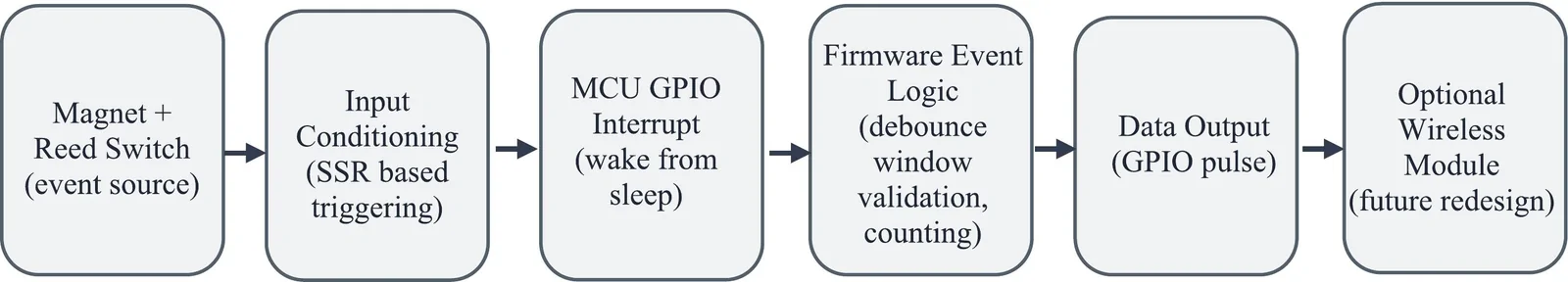
Signal flow and firmware block diagram.

The reed-switch transition is routed to the MCU external interrupt input through a solid-state switching element. In the embedded firmware, a 30 ms validation window is applied to suppress contact bounce; this time constant was selected to be consistent with the time scales obtained from the MATLAB/Simscape characterization of the pedal dynamics. The external interrupt wakes the MCU from deep sleep, enabling event validation and updating the count state. This signal flow and the firmware function blocks are summarized in Fig. 3.

Depending on deployment needs, validated events can be forwarded to an optional wireless interface or used to drive a physical output.

## 3 Design files summary

The complete set of design files required to reproduce and further develop the proposed wheel detector is provided in the associated open-source repository. These files include the mechanical CAD models, KiCad source files, fabrication outputs, enclosure geometry, firmware source code, and validation scripts. A summary of the available design files, file formats, licenses, and repository locations is provided in Table 3.

**Table 3:** Design files summary.

**Design file name**	**File type**	**Open source license**	**Location of the file**
Mechanical_STEP.zip	CAD files // STEP	CC BY 4.0	Source File Repository
Electronics_KiCad_Source.zip	KiCad source files	CC BY 4.0	Source File Repository
Electronics_Gerber_and_Drill.zip	Gerber + drill files	CC BY 4.0	Source File Repository
PCB_Enclosure.zip	CAD files // STEP	CC BY 4.0	Source File Repository
Firmware_Source.zip	Source code	CC BY 4.0	Source File Repository
Validation_Scripts_and_Data.zip	MATLAB, Python, CSV data	CC BY 4.0	Source File Repository

## 4 Bill of materials summary

The main mechanical, electronic, and enclosure components required to construct the prototype are summarized in Table 4. The table provides the component designation, quantity, approximate unit and total costs, sourcing information, and material or component type, while the complete detailed BOM files are available in the source repository.

**Table 4:** Bill of materials summary.

**Designator**	**Component**	**Quantity**	**Cost per unit [€]**	**Total cost [€]**	**Source of materials**	**Material Type**
Mechanical System	See separate BOM in repository	1	450	450	Source file repository	Al 6063 T6
C1-C2	Ceramic capacitor, 1 uF	2	0.73	1.46	https://cutt.ly/mtIGRDp7	Passive component
D1-D2	Diode, 1 N4001	2	0.10	0.20	https://cutt.ly/QtIGR8wE	Semiconductor
R1	Resistor, 2 k4	1	0.34	0.34	https://cutt.ly/btIGTivm	Passive component
R2-R3-R5	Resistor, 10 k	3	0.09	0.27	https://cutt.ly/htIGTzSo	Passive component
R4	Resistor, 1 k	1	0.28	0.28	https://cutt.ly/StIGFpF8	Passive component
RL1	Relay, G2RL-2-DC12	1	2.98	2.98	https://cutt.ly/3tIGTUDJ	Electromechanical component
S1	Terminal block, 3.81 mm 6 pin	1	3.04	3.04	https://cutt.ly/PtIGTCy7	Connector
U1	LDO, MCP1702-3302E/TO	1	0.56	0.56	https://cutt.ly/ptIGT8JV	Semiconductor
U2	Solid state relay, CPC1593G	1	2.98	2.98	https://cutt.ly/otIGYa23	Semiconductor
U3	MCU, NUCLEO-L432KC	1	10.15	10.15	https://cutt.ly/UtIGYGpq	Development board
Q1	Transistor, 2 N2222	1	3.17	3.17	https://cutt.ly/2tIGYMH5	Semiconductor
M3 Screws	M3 screw, 5 mm	4	0.10	0.40	https://cutt.ly/qtIGGKWi	Fastener
Printed circuit board (PCB)	See separate BOM in repository	1	0.40	0.40	https://cutt.ly/OtIGUkW8	FR4 PCB
PCB enclosure	See separate BOM in repository	1	25	25	Source file repository	Enclosure
**Estimated total cost**				**501.23**		

## 5 Build instructions

The wheel detector can be assembled using the mechanical, electronic, enclosure, and firmware files provided in the source file repository. The mechanical STEP files define the geometry of the pedal, housing, supporting components, and associated mounting parts, while the KiCad source files, Gerber files, and firmware source files are provided for reproduction of the electronic subsystem.

### 5.1 Mechanical assembly

The mechanical components should first be manufactured according to the STEP files provided in the repository. The main mechanical components and their relative arrangement are shown in Fig. 1. The pedal is installed inside the housing and constrained by the lateral bearings so that its motion is predominantly vertical. Two identical compression springs are positioned beneath the pedal and installed with a preload of 24 mm.

The permanent magnet is installed in the pedal, while the reed sensor is mounted at the base of the housing and oriented toward the magnet, as shown in Fig. 1. The vertical position of the reed sensor can be adjusted within the available 5 mm adjustment range to obtain the required magnet–reed switching geometry. After assembly, the pedal should move freely along its guided vertical path and return to its initial position under spring force without mechanical interference.

### 5.2 Electronic assembly

The electronic control board can be reproduced using the KiCad source files or the Gerber and drill files provided in the repository. The circuit architecture and the main power-regulation and reed-input conditioning connections are shown in Fig. 2. Electronic components should be assembled according to the provided schematic and the bill of materials.

After component assembly, the control board is installed inside the PCB enclosure, and the reed-switch input, power-supply, and detector-output connections are made through the corresponding terminal connections. The completed electronic subsystem is designed for an input supply range of 3.7–13 VDC.

### 5.3 Firmware installation

The firmware source code provided in the repository is programmed to the STM32L432KC-based controller using the standard STM32 programming interface. The functional signal flow from the reed-switch event source to the MCU interrupt, firmware validation logic, and detector output is summarized in Fig. 3.

The reed-switch signal is configured as an external interrupt source that wakes the microcontroller from deep sleep when a wheel-passage event occurs. The firmware applies a 30 ms validation window after each detected transition to suppress contact bounce and repeated triggering. After programming, detector operation should be checked manually by moving the pedal through the reed-switch activation position and confirming that a single validated output event is generated.

## 6 Operation instructions

The assembled wheel detector is intended for straightforward field deployment and low-power wheel-passage monitoring. Operation consists of installing the mechanical detector on the rail, connecting the electronic control unit, verifying the reed-switch triggering geometry, and confirming correct single-event generation before monitoring begins. The main steps for system setup, wheel-passage detection, and operational checking are described below.

### 6.1 System setup and installation

**Fig. 4:**
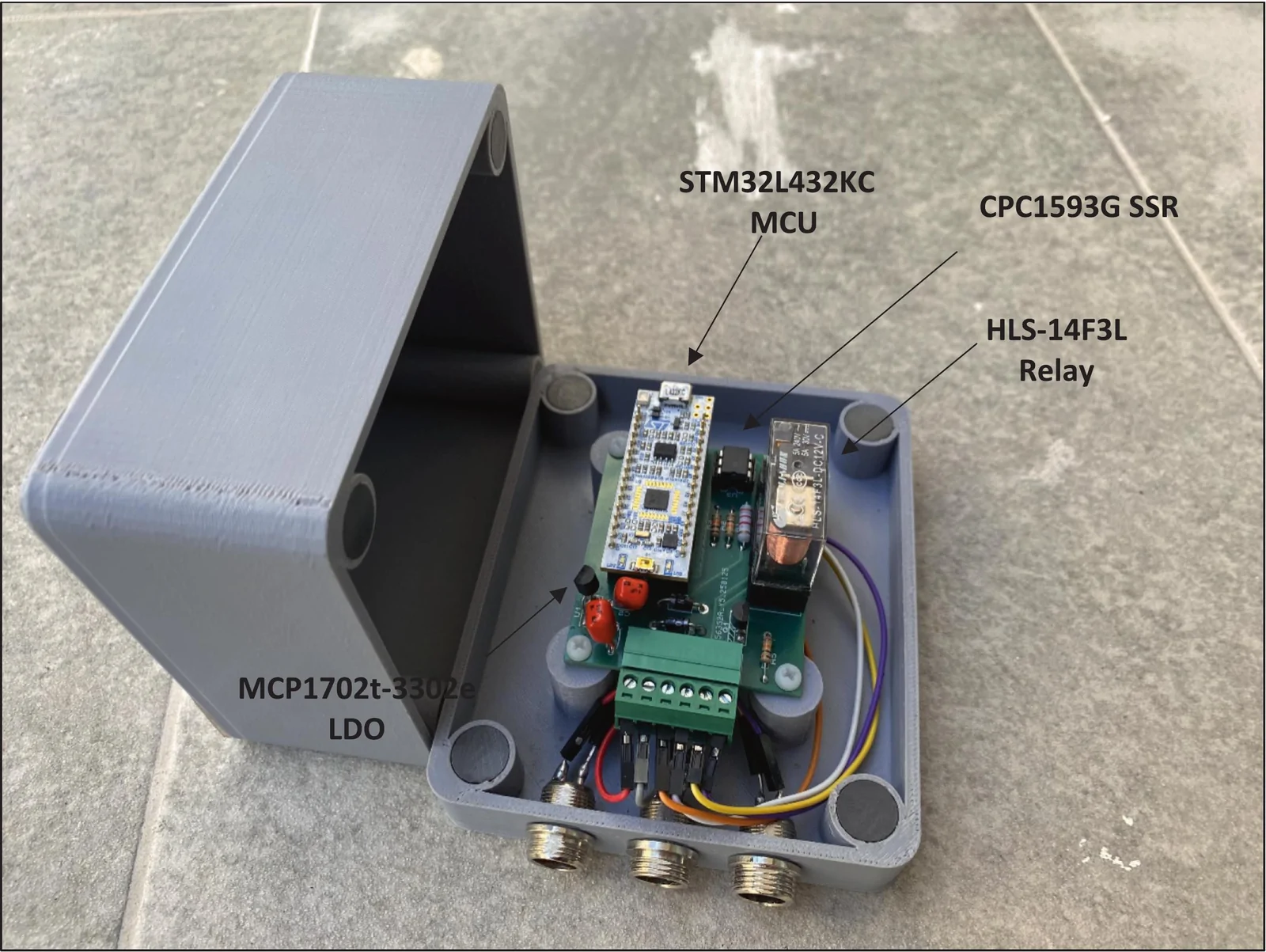
Physical prototype of the wheel node electronic control unit.

Fig. 4 shows the physical prototype of the electronic control unit developed for the wheel detector node. The assembled control board includes the microcontroller, power-regulation stage, reed-switch interface, relay output, and terminal connections required for field operation. The control board is installed inside its enclosure before connection to the mechanical detector assembly.

**Fig. 5:**
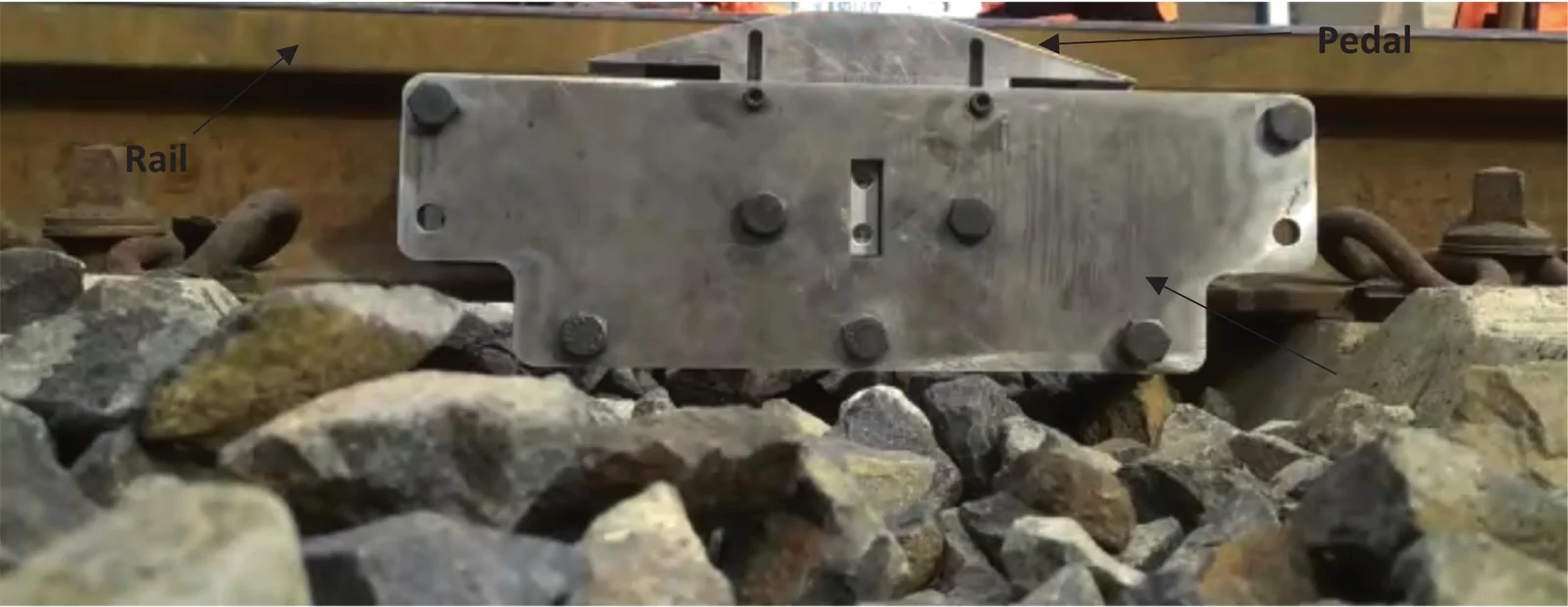
Field installation of the proposed wheel detector.

The assembled wheel detector is installed on the rail as shown in Fig. 5. During installation, the pedal surface is positioned 14 mm below the top of the rail head to ensure contact with the wheel flange. The detector should be securely mounted while allowing the pedal to move freely along its guided vertical path without mechanical interference.

The reed sensor position can be adjusted within the available 5 mm vertical adjustment range to obtain the required switching geometry. The magnet–reed gap is adjustable between 22 and 27 mm. After mechanical installation, the electronic control unit is connected to the reed-switch output and supplied from a 3.7–13 VDC power source.

Before operation, the pedal should be manually depressed and released to verify free vertical movement, spring-assisted return to its initial position, and reliable reed-switch activation. The detector output should also be checked to confirm that a single validated event is generated for each manual activation.

### 6.2 Wheel detection

During normal operation, the detector remains in its low-power state until a wheel flange contacts the pedal. The downward displacement of the pedal reduces the distance between the permanent magnet and the reed sensor until the sensor is activated and a switching event is generated.

The resulting signal is transferred through the input-conditioning stage to the microcontroller interrupt input according to the signal flow shown in Fig. 3. The interrupt wakes the microcontroller from deep sleep, after which the firmware applies a 30 ms validation window to suppress contact bounce and rebound-related repeated triggering. Following validation, a single wheel-passage event is registered and the corresponding detector output is generated.

After the wheel flange leaves the detector, the compression springs return the pedal to its initial position, restoring the magnet–reed separation and preparing the detector for the next wheel passage. In the single-detector configuration used in this study, each valid detection event corresponds to one wheel-flange passage on the instrumented rail.

### 6.3 Operational check

Correct operation can be verified by comparing the generated detector events with observed wheel passages. The detector should generate one validated output event for each wheel-flange passage without repeated triggering during a single wheel event. Any irregular response should first be checked by verifying free pedal movement, correct spring return, the magnet–reed alignment, and the electrical connections between the mechanical detector and the electronic control unit.

## 7 Validation and characterization

### 7.1 Numerical characterization

In the proposed wheel node prototype, the interaction between the train wheel and the pedal mechanism is explained by contact mechanics; the vertical displacement of the pedal via springs, the magnet entering the detection area of the reed sensor during this process, and the pedal returning to its original position as the wheel moves forward are described by a dynamic model.

**Fig. 6:**
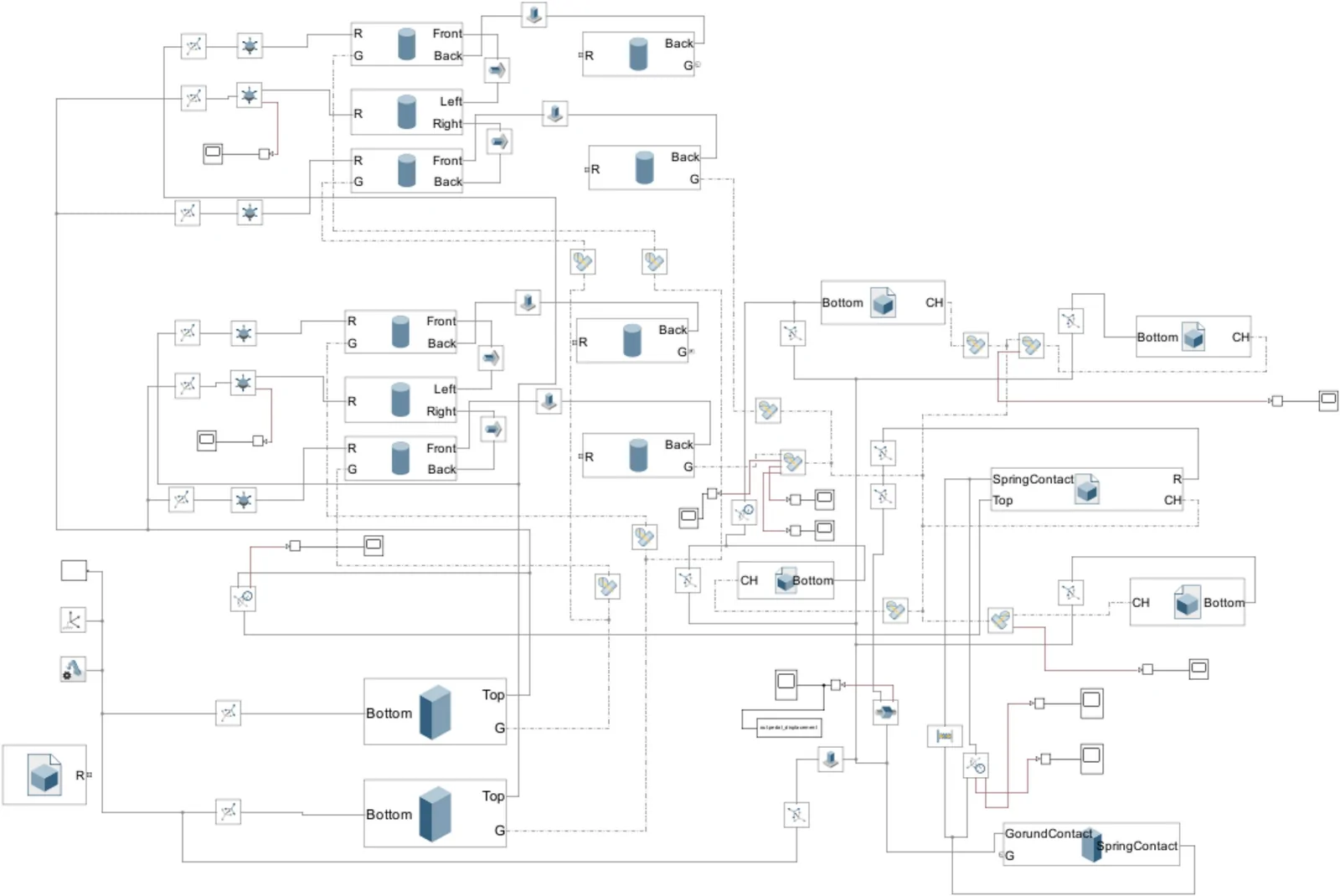
MATLAB/Simscape model.

To characterize the gross dynamic behavior of the pedal mechanism, a Simscape model was created (see Fig. 6). Sliding mechanical contact between the wheel flange and the pedal was used to impose a demanding momentum-transfer and rebound condition. The model examined the time-dependent behavior of the pedal–spring–magnet–sensor interaction at different wheel speeds, with particular emphasis on pedal return and damping characteristics. The Simscape formulation treats the pedal–spring subsystem as a lumped-parameter system and assumes that the pedal undergoes rigid-body motion at the assembly scale. Consequently, internal elastic or plastic stress-wave propagation and very short-duration local micro-separation at the contact interface are not resolved. Within this scope, the model was used to assess repeated magnet–reed crossings associated with gross pedal rebound and to support the selection of the digital validation interval applied to the microcontroller input.

The contact between the wheel flange and the pedal was modeled using Hertzian contact theory. Since the pedal exceeds the elastic limit at higher speeds, this study additionally includes an elastoplastic indentation analysis. The risk of local plasticity at the pedal tip was minimized by using a preloaded spring and by appropriate selection of the pedal material.

To improve the dynamic behavior of the pedal during wheel contact and immediately after release, spring preloading was applied. The preload reduces the tendency for rebound after the initial impact by shifting the response from a lightly underdamped oscillatory behavior toward a more controlled compression-dominated motion, thereby shortening the return-to-equilibrium time. As a result, the amplitude and duration of post-contact oscillations are reduced, minimizing repeated magnet–reed passages and multiple triggering events, and thus enabling more stable single-event generation. This also provides a safe and repeatable digital filtering window.

**Fig. 7:**
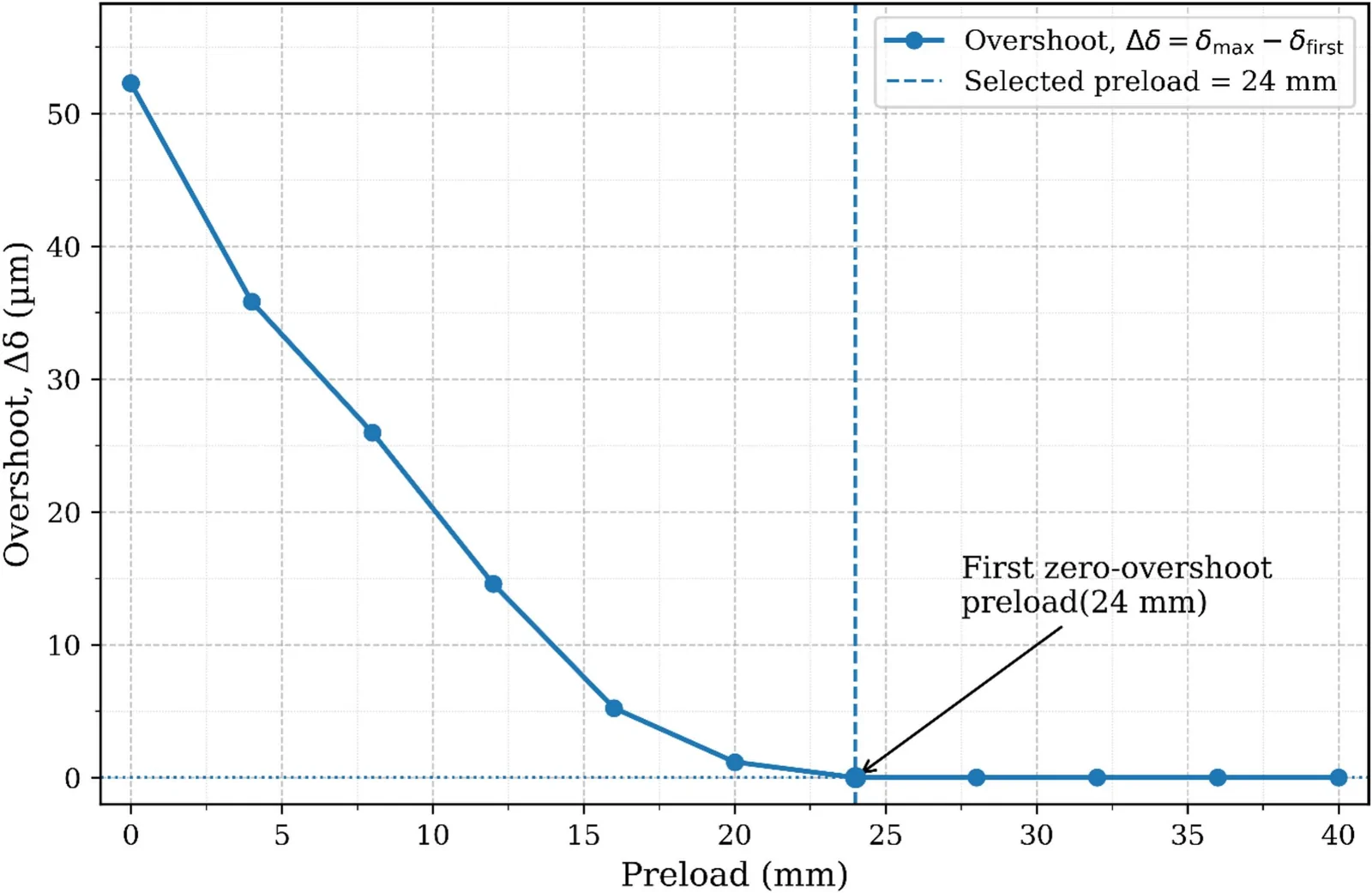
Overshoot based on spring preload at 90 km/h wheel speed.

The preload value was selected based on the overshoot phenomenon. In this study, overshoot was defined as the difference between the maximum penetration and the first-contact penetration, $\Delta \delta =\delta_{\max}-\delta_{\mathrm{first}}$. This quantity represents the extent of additional penetration occurring after the initial contact due to inertial and elastic transient effects (see Fig. 7). High overshoot values indicate a more pronounced dynamic transient response, higher instantaneous contact severity, and less controlled contact characteristics. The results showed that the overshoot decreased monotonically with increasing preload and reached zero for the first time at a preload of 24 mm. Therefore, 24 mm was selected as the minimum preload capable of completely eliminating secondary penetration. Higher preload levels were not preferred, since they provided no further improvement in overshoot suppression.

Al 6063-T6 alloy is used for the wheel detector to provide a deliberate mechanical fuse behaviour at the wheel–pedal interface. The wheel material is significantly stronger and harder than the detector structure; therefore, under accidental overloads, misalignment, or unexpected impact conditions, it is preferable for any irreversible deformation to occur in the replaceable detector components rather than in the wheel. Using a material with a lower yield strength than the wheel promotes this hierarchy [16]. The detector can plastically deform in a controlled manner to absorb energy and limit peak contact stresses, while the wheel remains within its elastic regime. In addition, the lower density of Al 6063-T6 reduces the moving mass of the pedal assembly, which helps decrease inertia-driven rebound and supports faster reset after a wheel passage—both of which contribute to stable single-event generation.

The Hertzian approach allows the load–indentation relationship to be expressed in closed form and thereby provides a physical basis for estimating the contact stiffness. In the present Simscape implementation, the stiffness parameter (k_n_) of the linear penalty-contact block was calibrated using the tangent stiffness of the Hertzian force law at the maximum expected load. A single operating-point value was selected to retain a transparent and computationally stable lumped-parameter model suitable for preload and damping sensitivity studies, while matching the local slope of the Hertzian response near the peak-load condition. This operating-point calibration is an approximation and does not reproduce the continuously displacement-dependent stiffness of the complete Hertzian contact law.

For two elastic bodies, the equivalent elastic modulus $E^{*}\;$ and the equivalent radius of curvature $R^{*}\;$ (under local spherical approximation) are given by:

(1) $$\frac{1}{E^{*}}=\;\frac{1-\nu_{1}^{2}}{E_{1}}+\;\frac{1-\nu_{2}^{2}}{E_{2}}\left[17\right]$$

(2) $$\frac{1}{R^{*}}=\;\frac{1}{R_{1}}+\;\frac{1}{R_{2}}\left[17\right]$$

where $E_{i}$ and $\nu_{i}$ denote the Young’s modulus and Poisson’s ratio of the materials, and ${\;R}_{i}\;$ represent the local radii of curvature at the contact point. In the wheel flange–pedal interface, a local spherical approximation is applied using the pedal tip geometry and the local curvature of the wheel flange. The Hertzian pressure–indentation relationship for spherical contact is expressed as:

(3) $$P\left(\delta \right)=\;\frac{4}{3}\;E^{*}\;\sqrt{R^{*}}\;\delta^{\frac{3}{2}}\left[17\right]$$

The instantaneous (tangential) contact stiffness, i.e., the linearized stiffness k_n_ at the operating point, is defined as the derivative of force with respect to indentation:

(4) $$k_{n\;}\left(\delta \right)=\;\frac{\mathrm{dF}}{d\delta }\left[17\right]$$

(5) $$k_{n\;}\left(\delta \right)=2\;E^{*}\;\sqrt{R_{\mathrm{eff}}}\;\sqrt{\delta }\left[17\right]$$

Solving the force equation for indentation yields:

(6) $$\delta ={\left(\;\frac{3\;F\;}{4\;E^{*\;}\sqrt{R^{*}}}\right)}^{\frac{2}{3}}\left[17\right]$$

In the Simscape model, selecting the linear equivalent normal stiffness for the physical contact parameters plays an important role in determining the oscillation amplitude of the mechanical response and the optimum range of the digital filtering window. This choice ensures that, at the operating point of the linear penalty-based contact model, the slope of the real (nonlinear) Hertzian contact behaviour is captured. Consequently, the return oscillation amplitude of the pedal, which is an important factor governing the risk of multiple reed-switch triggers, is controlled in a physics-based manner. The material selection of the pedal also directly affects the choice of the linear equivalent stiffness coefficient. In the wheel–pedal interaction, the linear equivalent stiffness is implemented as a penalty-based normal contact stiffness.

In the literature, the normal stiffness of wheel–rail contact in Hertzian-based models is reported to be approximately (2.0–9.6)×10^8^ N/m for loads in the 1–100 kN range, and the linear equivalent stiffness increases with increasing load. The linear equivalent stiffness values were optimized by minimizing the differences between the loads obtained in simulation and those predicted by the theoretical calculations [[17], [18]].

(7) $$\zeta =\;\frac{\left|ln\varepsilon \right|}{\sqrt{\pi^{2}+\;{\left(ln\varepsilon \right)}^{2}}}\;\left[19\right]$$

(8) $$C=2\;\zeta \;\sqrt{K\;m_{\mathrm{eq}}}\left[19\right]$$

In the simulation, the damping coefficient C was computed according to the damping ratio ζ, which depends on the theoretical linear equivalent stiffness K and the restitution coefficient ε, as given by Eqs. (7), (8), and then implemented in the model. The restitution coefficient ε was set to 0.95 to represent a deliberately demanding rebound condition. This prescribed value was not coupled to the plastic energy dissipation calculated in the elastoplastic model; in practice, local yielding of the Al 6063-T6 pedal would be expected to reduce the effective restitution coefficient and the resulting rebound response [19].

The pedal’s vertical travel was limited to 26 mm, thereby preventing contact within the sensor assembly. The upper and lower stops providing this limitation were treated as metal-to-metal contacts in the design and were modelled accordingly. In the prototype, these stops are also suitable for implementation with an elastomer material to introduce additional damping.

Guidance for vertical motion of the pedal is provided by two bearings on each side of the pedal, which run in contact with the lateral walls of the base. This contact contributes to damping of the spring oscillation. Considering experimental studies reporting the low-damping characteristics of bearing-guided mechanisms, a structural damping ratio of (ξ = 0.5%) was adopted as a representative nominal value for the pedal–spring subsystem. This value is close to the 0.7% level reported for similar bearing-guided configurations and its influence was subsequently examined through the damping sensitivity analysis. Accordingly, the damping coefficient C was calculated as 12.85 N·s/m and used in the simulation model [[20], [21]].

**Fig. 8:**
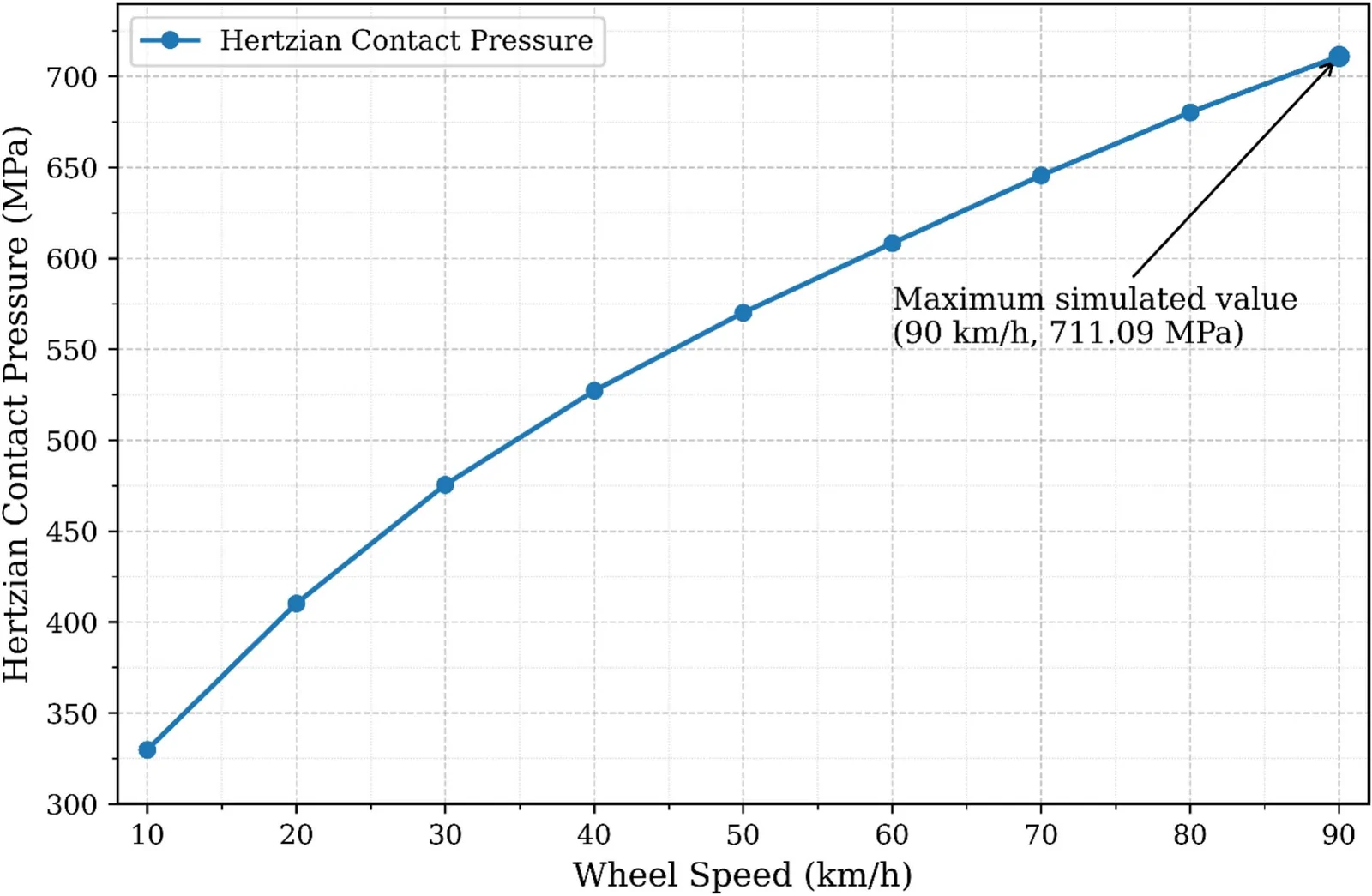
Hertzian contact pressure vs. wheel speed under the fully elastic contact assumption.

Fig. 8 shows the variation of Hertzian contact pressure with wheel speed under the fully elastic contact assumption. The results indicate that the contact pressure increases monotonically from 329.83 MPa at 10 km/h to 711.09 MPa at 90 km/h. However, the increase is clearly nonlinear, with a progressively decreasing slope at higher speeds. This trend is consistent with the nonlinear nature of Hertzian contact, where the contact area increases with normal load, preventing the pressure from rising linearly with impact severity [22]. Therefore, the obtained curve represents an elastic reference baseline for the wheel–pedal interaction. This baseline is particularly useful for identifying the speed regime in which the aluminum pedal material may deviate from purely elastic behavior and enter an elastic–plastic contact state.

(9) $$P_{0,y}=1.6\;\sigma_{y}\left[17\right]$$

Equation (9) expresses the approximate relationship between the maximum Hertzian contact pressure at the onset of yielding, P_0,y_ and the uniaxial yield strength of the material, σ_y_ [22]. According to EN 13262, the minimum yield strength of the lowest-strength railway wheel rim steel grade is about 500 MPa, corresponding to an onset-of-yield Hertzian pressure of approximately 800 MPa [16]. EN 755–2 gives a 0.2% proof stress of about 170 MPa for common T6 extruded product conditions, which corresponds to an onset-of-yield Hertzian pressure of approximately 272 MPa [23]. Therefore, once this threshold is exceeded, the 6063-T6 pedal can no longer be described by a purely elastic Hertzian contact state and the contact enters an elastic–plastic regime, while the wheel rim is still expected to remain elastic.

(10) $$H\approx 3\;\sigma_{y,Al}\left[24\right]$$

As plastic deformation develops, the aluminium surface flattens and the real contact area increases. Under these conditions, the mean interfacial pressure approaches a hardness-like limit described by the Tabor relation, which is approximately three times the yield strength of the deforming alloy (see Equation (10)). For Al 6063-T6, this corresponds to a value of about 510 MPa. This Tabor-limited pressure is well below the approximate wheel-rim yielding threshold discussed above. Hence, plastic deformation is expected to localize primarily in the aluminium pedal rather than in the wheel flange. As the contact area expands during plastic deformation, further increases in normal load are accommodated mainly by additional plastic indentation and contact-area growth, rather than by unlimited growth of elastic Hertzian peak pressure. This behavior supports repeated wheel passages while avoiding plastic deformation of the wheel flange [16], [23], [24].

According to UIC standards, the minimum wheelbase for passenger and freight vehicles with two-axle bogies is 2000 mm and 1800 mm respectively. For railway vehicles with more than two axles, the minimum wheelbase is set at 1500 mm [10]. Therefore, in the Simscape model, the wheelbase is set to 1500 mm.

**Fig. 9:**
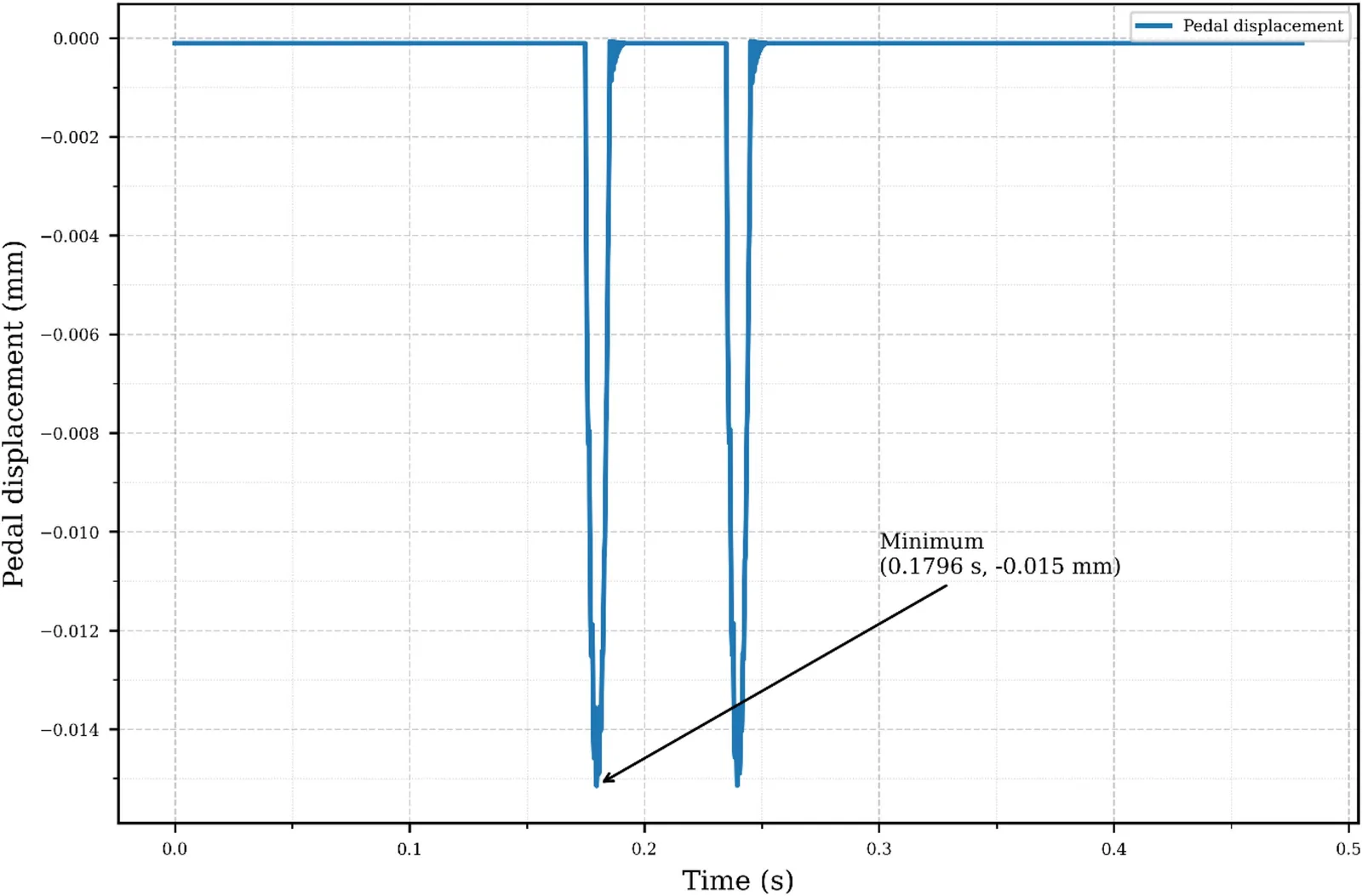
Pedal displacement response at 90 km/h.

Fig. 9 shows the displacement of the pedal after contact with the flanges of the wheels of a two-axle bogie traveling at 90 km/h. The pedal displacement response demonstrates a dynamically stable contact behavior.

The two pronounced displacement valleys correspond to the two axle-related wheel-passage events of the bogie. In each event, the pedal undergoes a rapid downward motion and then returns to its initial position within a short time. Post-contact oscillations remain limited, and the pedal becomes fully damped within 17.076 ms. Within the adopted linearized contact model, this result indicates that the applied preload suppresses gross rebound and promotes a more controlled compression-dominated response. Furthermore, the predicted settling time remains below the previously defined 30 ms digital filtering window, supporting the adequacy of the selected 30 ms validation window for suppressing rebound-related repeated triggering within the adopted modelling assumptions. Therefore, the 24 mm preload provides a suitable design point in terms of both mechanical stability and reliable single-event generation.

Table 5 compares the fully damped pedal response time with the wheelbase travel time for different wheel speeds. The results show that, although both quantities decrease with increasing speed, the pedal response time remains lower than the corresponding wheelbase travel time throughout the investigated range. This indicates that the pedal mechanically settles before the next wheel-passage event occurs. Even under the most critical case of 90 km/h, the pedal becomes fully damped within 17.076 ms, while the wheelbase travel time is 60 ms. Therefore, the proposed detector maintains adequate temporal separation between consecutive wheel events over the investigated speed range.

**Table 5:** Pedal response time.

**Wheel Speed (km/h)**	**Fully Damped Response Time (ms)**	**Wheelbase Travel Time (ms)**
10	185.77	540
20	50.35	270
30	35.28	180
50	24.15	108
60	22.42	90
75	20.30	72
90	17.07	60

Furthermore, a sensitivity analysis was performed to assess the influence of the assumed structural damping ratio on the pedal settling time at 90 km/h. While the nominal model used a damping ratio of 0.5%, additional simulations were carried out for 0.3% and 0.7%, corresponding to a range broader than the ±20% variation requested in the review. The fully damped response times obtained were 21.17 ms, 17.08 ms, and 16.77 ms for damping ratios of 0.3%, 0.5%, and 0.7%, respectively. These results show that, although the settling time varies with damping, the response remains below the selected 30 ms digital filtering window in all investigated cases.

On the other hand, in order to evaluate the plastic deformation of the aluminium pedal and to determine whether the resulting residual indentation could affect the mechanical reference levels of the wheel detector, the contact was analysed using a reduced-order 2D elastoplastic finite-element indentation model. To evaluate whether residual plastic indentation would accumulate under repeated wheel passages, the reduced-order elastoplastic indentation model was extended to repeated load–unload cycles under the worst-case 90 km/h contact condition. In this cyclic analysis, the plastic strain history, stress state, and residual displacement field were retained between successive cycles. Therefore, each new wheel passage was applied to the already plastically accommodated and strain-hardened pedal surface, rather than to an undeformed material state.

The Hertzian solution was used only as an elastic reference envelope for defining the peak-pressure level and equivalent loaded width. The maximum normal contact force obtained from the Simscape model was F_ext_ = 136.849 kN, while the corresponding maximum Hertzian pressure was P_0_ = 711.09 MPa. The equivalent contact patch area was estimated from the integrated elliptical Hertz pressure relation $F=\frac{2}{3}P_{0}A$, giving A = 288.67 mm^2^. This area was converted into an equivalent circular contact radius of 9.59 mm, corresponding to a 19.17 mm loaded width in the reduced 2D plane-strain model [22].

The pedal was represented as a 2D plane-strain Al 6063-T6 stion. The upper contact region was loaded using a Hertz-like pressure distribution, while all external boundaries except the loaded upper surface were fixed to represent a conservative support condition. The use of a 2D plane-strain formulation was selected because the quantity of interest was the residual vertical indentation, rather than a full three-dimensional rolling/sliding wear pattern. A 3D indentation model could provide additional spatial details of the contact patch and edge effects, but it would require further assumptions regarding the local flange profile, local pedal curvature, and evolving three-dimensional contact geometry. For the present purpose, the 2D formulation is used as a reduced-order estimate of the vertical indentation relative to the 5 mm triggering allowance.

The finite-element formulation was based on the incremental solution of the nonlinear equilibrium equations. In the absence of body forces, the continuum equilibrium problem can be written as [25]

(11) ∇·σ=0[25]

where σ is the stress tensor. After finite-element discretization, the nonlinear equilibrium equation at each load step becomes

(12) $$R\left(d\right)\;=\;F_{\mathrm{ext}}-\;F_{\mathrm{int}}\left(d\right)\;=\;0\left[25\right]$$

where d is the nodal displacement vector, F_ext_ is the external load vector, and F_int_ is the internal force vector. The internal force vector is obtained from the stress field as

(13) $$F_{\mathrm{int}}=\;\Sigma \;\int \;B^{T}\;\sigma \;d\Omega \left[25\right]$$

where B is the strain–displacement matrix and Ω is the element domain. The nonlinear system was solved incrementally using the Newton–Raphson method,

(14) $$K_{t}\Delta d=R$$

where K_t_ is the tangent stiffness matrix and Δd is the displacement correction vector.

For the 2D plane-strain condition, the strain field was written as

(15) ε=Bd

with the in-plane strain vector

(16) $$\varepsilon \;=\;\left[\;\varepsilon_{\mathrm{xx}},\;\varepsilon_{\mathrm{yy}},\;\gamma_{\mathrm{xy}}\;\right]\left[25\right]$$

and the plane-strain constraint

(17) $$\varepsilon_{\mathrm{zz}}\;=\;0\left[25\right]$$

The Al 6063-T6 pedal was modelled using a small-strain J2 von Mises elastoplastic formulation with isotropic hardening, following the standard computational plasticity framework [26]. This constitutive law was used as a baseline representation of initial yielding and strain hardening. It does not include a kinematic backstress term and therefore does not explicitly reproduce the Bauschinger effect or progressive plastic strain accumulation associated with ratcheting under cyclic loading. The stress–strain relation was written as

(18) $$\sigma \;=\;D\;\left(\;\varepsilon \;-\;\varepsilon_{p}\;\right)\left[26\right]$$

where D is the elastic stiffness matrix and ε_p_ is the plastic strain. The von Mises equivalent stress was calculated from the deviatoric stress s as

(19) $$\sigma_{\mathrm{eq}}\;=\;\sqrt{\;\frac{3}{2}\;s\;:\;s\;}\left[26\right]$$

With

(20) $$s\;=\;\sigma \;-{\;\sigma \;}_{m}\;I\left[26\right]$$

where σ_m_ is the mean normal stress. The yield function was defined as

(21) $$f\;=\;\sigma_{\mathrm{eq}}\;-\;\left(\;\sigma_{y}\;+\;H\;\varepsilon_{p,eq}\;\right)\;\leq \;0\left[26\right]$$

where σ_y_ is the initial yield stress, H is the isotropic hardening modulus, and ε_p,eq_ is the accumulated equivalent plastic strain and f is isotropic hardening function. If f ≤ 0, the response remains elastic. If f > 0, the stress state is corrected using the radial return-mapping procedure. The plastic multiplier increment was computed as

(22) $$\Delta \gamma \;=\;f_{\mathrm{trial}}\left(3G\;+\;H\right)\left[26\right]$$

where G is the shear modulus. The accumulated equivalent plastic strain was then updated as

(23) $${\varepsilon \;}_{p,eq}^{\mathrm{new}}=\;{\varepsilon \;}_{p,eq}^{\mathrm{old}}+\;\Delta \gamma \left[26\right]$$

For each wheel-passage cycle, the pressure was incrementally increased to the maximum value and then completely removed. The pressure distribution over the equivalent loaded width was defined as

(24) $$P_{x}=\;\alpha \;P_{0}\;\sqrt{1-{\left(\frac{x}{a}\right)}^{2}}\;for\;\left|x\right|\;\leq \;a\left[17,25\right]\;$$

where P_0_ is the maximum Hertzian pressure, a is half of the equivalent loaded width, and α is the load factor. During loading, α increases from 0 to 1; during unloading, α decreases from 1 to 0. The stress state, plastic strain history, and residual displacement field were retained between successive cycles. Therefore, each new wheel passage was applied to the already plastically accommodated and strain-hardened pedal surface, rather than to an undeformed material state.

After each unloading stage, the accumulated residual indentation was obtained from the remaining vertical displacement of the upper contact surface. This was evaluated as the maximum downward residual displacement in the loaded contact region:

(25) $$h_{N}\;=\;max\left(-u_{y,N}\right)\left[26\right]$$

where h_N_ is the residual indentation after cycle N and u_y,N_ is the residual vertical displacement after unloading. The incremental indentation per cycle was calculated as

(26) $${\Delta h}_{N}=h_{N}-h_{N-1}\left[26\right]$$

The cyclic model includes strain hardening but does not update the enlargement of the real contact area caused by plastic surface flattening. In the actual wheel–pedal interaction, the initially plastically deformed aluminium surface would be expected to become locally flatter and more conformal with the wheel-flange contact region. This would increase the real contact area and reduce the local contact pressure during subsequent passages. In contrast, the present model repeatedly applies the same Hertz-equivalent pressure envelope over the same loaded width in every cycle. This repeated-pressure assumption is therefore conservative with respect to further plastic accumulation, because it does not credit the pressure-reducing effect of contact-area growth after the initial bedding-in deformation.

Within the adopted isotropic-hardening formulation, the repeated-cycle analysis predicted a shakedown-like response. The residual indentation increased mainly during the first few cycles and then approached a stable value. In this analysis, shakedown was defined as the condition where the incremental residual indentation remained below 0.001 mm/cycle for five consecutive cycles. This criterion was satisfied after 9 load–unload cycles. At this stage, the predicted accumulated residual indentation was approximately 0.70 mm, while the final incremental indentation was only 8 × 10 − 6 mm/cycle. These results indicate that, within the adopted constitutive formulation and simulated cycle range, the residual indentation did not accumulate linearly. The predicted residual indentation remained below the 5 mm functional allowance reserved in the reed-switch triggering geometry.

**Fig. 10:**
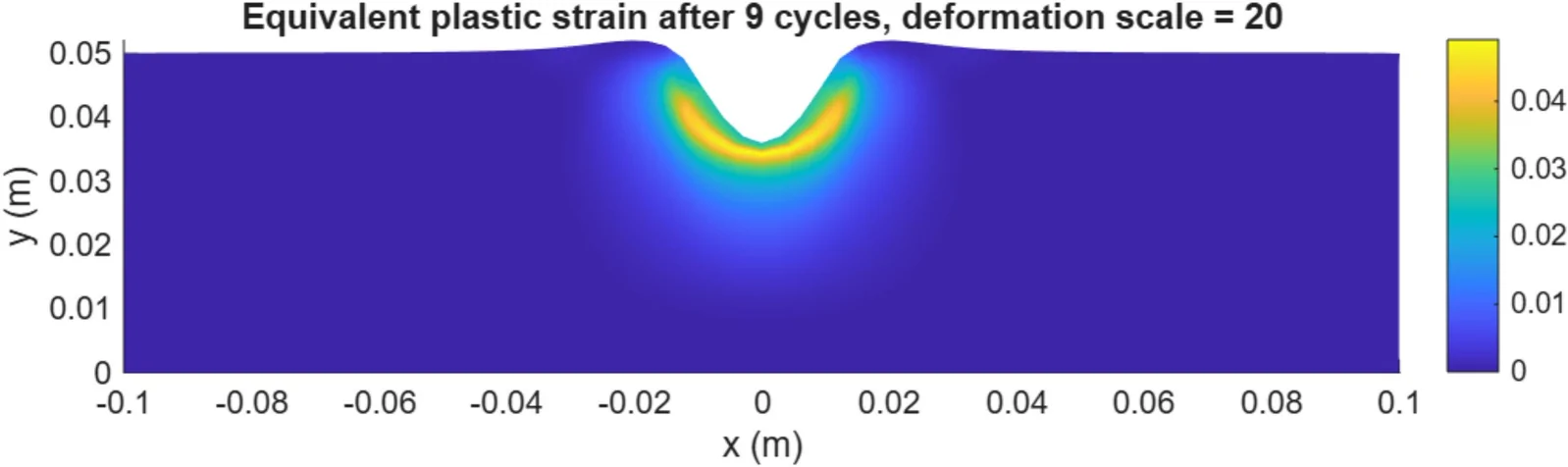
Equivalent plastic strain distribution in the Al 6063-T6 pedal after repeated load–unload cycles.

Fig. 10 shows the equivalent plastic strain distribution after the shakedown criterion was reached. This figure does not represent the indentation depth; instead, it shows the accumulated plastic strain field within the pedal section after unloading. The maximum equivalent plastic strain was approximately 0.0506, which is a dimensionless strain value. The plastic strain was concentrated in the upper contact region, particularly around the shoulders of the residual indentation, while the lower region of the pedal remained essentially unaffected. This localized strain field supports the intended mechanical-fuse behaviour: irreversible deformation is confined to the replaceable Al 6063-T6 pedal instead of spreading through the detector body or being transferred to the wheel flange.

### 7.2 Field validation

Field validation was performed under real railway operating conditions over the speed range of 0–90 km/h using a single railway vehicle with a known axle configuration. Because the same vehicle was used throughout the test, wheel-passage events on the instrumented rail could be verified by direct visual observation and compared with the detector output. In the present single-detector configuration, each valid event corresponds to one wheel-flange passage on the instrumented rail. In addition, the same wheel flanges were visually inspected after the test to identify possible deformation. Table 6 summarizes the observed detection performance for the 500 monitored wheel-passage events.

**Table 6:** Visual Test for Field Validation.

Parameter	Value/Observation
Test environment	Real railway field installation
Instrumented configuration	Single wheel detector mounted on one rail
Test vehicle	Single railway vehicle with known axle configuration
Speed range	0–90 km/h
Validation method	Direct visual observation of wheel passages and comparison with detector output
Total monitored wheel-passage events on the instrumented rail	500
Recorded valid detection events	500
Missed detections	0
False-positive detections	0
Post-test wheel-flange inspection	No visible wheel-flange deformation observed

## 8 Discussion and limitations

The proposed wheel detector was developed as a low-power, open-hardware, mechanically triggered monitoring node for non-safety–critical railway applications. Within this scope, its main contribution is not to replace certified train-detection infrastructure, but to provide a simple and field-deployable event-generation mechanism for temporary monitoring, auxiliary triggering, and low-cost experimental deployment.

Table 7 indicates that reed-switch-based and electromagnetic coil-based wheel-detection concepts are governed by different trade-offs rather than a simple hierarchy of superiority. Electromagnetic coil-based systems are more mature in established railway practice, but their performance is more strongly tied to analog signal quality, filtering strategy, and robustness against electromagnetic disturbance. By contrast, the reed-switch-based approach adopted in this study simplifies the event-generation path and is structurally more compatible with ultra-low-power, battery-operated, and open-hardware monitoring nodes. Its main constraints are instead associated with geometric consistency, rebound control, and environmental drift of the switching distance. Therefore, the present detector should be interpreted as a low-cost, monitoring-oriented alternative for non-safety–critical railway applications where simplicity, low standby power, and field deployability are prioritized. An additional practical limitation concerns the long-term switching durability of the reed-based sensing element under field operation. Although repeated-cycle reliability testing of magnetic contacts has been reported in the literature, the service life of reed-based sensing remains dependent on contact configuration, loading, and operating conditions. Accordingly, the long-term switching durability of the assembled detector should be established through accumulated field testing rather than inferred solely from nominal component-level behavior.

**Table 7:** Qualitative comparison between reed-switch-based and electromagnetic coil for wheel detection.

**Comparison aspect**	**Reed Switch**	**Electromagnetic Coil**
Sensing principle	Magnetic-contact switching triggered by relative magnet motion [27], [28]	Inductive/electromagnetic response generated by wheel passage through the sensing field [29], [30]
Signal type	Binary event-oriented output [28]	Analog signal requiring thresholding and signal processing [29]
Energy demand	Suitable for ultra-low-power, battery-oriented architectures [6]	Typically more dependent on continuous sensing electronics and signal-conditioning stages [29]
Dominant failure modes	Chatter, switching-distance drift, misalignment, contact ageing, temperature/vibration sensitivity [28], [31]	Threshold-crossing errors, EMI-induced disturbance, rail magnetization effects, signal distortion/noise [29], [30]
Stability	Geometric consistency, stable magnet–sensor gap, controlled rebound [28], [31]	Signal-to-noise ratio, filtering strategy, robust decision thresholds [29], [30]
EMI behaviour	Lower dependence on inductive signal generation, but still dependent on interface electronics [28]	More directly affected by electromagnetic disturbance and magnetic-field interference [30]
False-detection tendency	Mainly related to mechanical rebound, chatter, or magnetic switching inconsistency [28]	Mainly related to signal disturbance, threshold instability, and environmental electromagnetic effects [30]
Environmental sensitivity	Switching distance and hysteresis can vary with environmental conditions [27]	Harsh environments may require signal-conditioning and filtering to preserve reliability [29], [30]
Maintenance	Simple architecture, replaceable low-cost parts, suitable for temporary or monitoring-oriented deployment [6], [32]	More mature and established in railway practice, but generally more infrastructure-dependent and application-specific [3], [32]

Beyond the qualitative positioning given in Table 7, the results of the present study indicate that the proposed detector achieves a practically consistent balance between mechanical response, triggering robustness, and low-power event generation within its intended monitoring scope. The preload optimization showed that 24 mm is the minimum value capable of eliminating secondary penetration, thereby suppressing rebound-driven contact transients at the sensing interface. This design choice is further supported by the linearized dynamic model, which predicted full damping within 17.076 ms at 90 km/h, while the selected digital filtering window was 30 ms. Moreover, the damping sensitivity analysis demonstrated that even under reduced structural damping, the response time remained below this filtering threshold. In parallel, within the adopted reduced-order 2D plane-strain formulation, the cyclic elastoplastic analysis predicted a shakedown-like response, with the residual vertical indentation approaching 0.70 mm, remaining well below the 5 mm triggering allowance of the reed-switch arrangement. Taken together, these findings suggest that, within the investigated operating envelope, the dominant mechanical design parameters remain compatible with reliable single-event generation and do not indicate an immediate loss of triggering functionality.

One of the limitations of the wheel detector node concerns the geometric reduction used in the elastoplastic indentation analysis. The finite three-dimensional Hertzian contact patch was mapped onto an equivalent pressure distribution applied as a continuous out-of-plane stripe in the 2D plane-strain model. The plane-strain condition suppresses out-of-plane deformation and therefore imposes greater kinematic constraint on the pedal material than a finite three-dimensional indentation field. For a given mapped pressure distribution, this additional constraint tends to delay local plastic flow and may therefore overestimate the contact pressure or load required for the onset of yielding. Conversely, replacing the finite three-dimensional contact patch with a continuous loading stripe removes the out-of-plane spatial decay of the applied pressure and may broaden the predicted stressed and plastically affected region after yielding. Consequently, the influence of the dimensional reduction is not uniformly conservative with respect to the final residual indentation: yield initiation may be delayed, whereas the spatial extent of the post-yield plastic zone may be overestimated. The calculated residual indentation should therefore be interpreted as a reduced-order, model-dependent estimate of the vertical plastic response. A fully three-dimensional wheel-flange–pedal contact model would be required to quantify the net influence of these competing geometric effects.

Another constitutive limitation arises from the exclusive use of isotropic hardening in the cyclic elastoplastic model. Isotropic hardening uniformly expands the yield surface as accumulated plastic strain increases and therefore provides a useful baseline for initial yielding and early cyclic accommodation. However, it does not represent translation of the yield surface or the associated Bauschinger effect. Although the externally applied wheel-contact pressure is compressive and is removed rather than fully reversed during each cycle, local residual stresses and multiaxial stress redistribution during unloading and reloading may produce partial stress-path reversals in the indentation region. Under such conditions, a purely isotropic formulation may predict shakedown more rapidly than a kinematic or mixed-hardening model and may underestimate continued cycle-by-cycle accumulation of residual plastic deformation. Accordingly, the predicted stabilization after 9 cycles and the residual indentation of approximately 0.70 mm should not be interpreted as verified long-term upper bounds. If ratcheting occurs in service, continued indentation could progressively reduce the available 5 mm triggering allowance. A mixed isotropic–kinematic formulation calibrated using cyclic material data for Al 6063-T6 would be required to evaluate the Bauschinger effect, ratcheting, and long-term cyclic accumulation more realistically.

The dynamic model is also subject to the operating-point linearization used for the normal contact stiffness. In a Hertzian contact, the normal force varies with penetration and the corresponding tangent stiffness therefore changes continuously. The present Simscape model instead applies a constant stiffness calibrated at the maximum expected load. This choice provides a transparent lumped-parameter representation and captures the local Hertzian slope near the peak-load condition, which is relevant to the maximum contact force and gross rebound response. However, the peak-load stiffness is higher than the Hertzian tangent stiffness during the lower-penetration portions of contact. The linearized model may therefore represent the contact as overly stiff during engagement and separation, potentially shortening the predicted impact duration and increasing the transient oscillation frequency. Accordingly, the calculated settling time should not be regarded as inherently conservative solely because of the stiffness linearization and may be underestimated relative to a fully nonlinear displacement-dependent contact law. The low structural damping and high restitution coefficient adopted in the model were intended to retain a demanding rebound condition, but the net conservatism of these competing assumptions cannot be quantified without a directly coupled nonlinear contact comparison. The reported 17.08 ms settling time should therefore be interpreted as an operating-point-based model prediction supporting the selected 30 ms firmware window, rather than as a rigorously established upper bound.

A related limitation is the physical decoupling between the prescribed restitution coefficient in the lumped dynamic model and the plastic energy dissipation predicted by the elastoplastic indentation model. The restitution coefficient was imposed as a system-level modelling parameter and was not updated as a function of local yielding or accumulated plastic work. In the actual wheel–pedal interaction, plastic deformation of the Al 6063-T6 contact region would dissipate part of the impact energy and would therefore tend to reduce the effective restitution coefficient and the macroscopic rebound response. The adopted high value intentionally neglects this additional dissipation and thereby retains a demanding rebound condition for evaluating rebound-related multiple triggering and the 30 ms firmware validation window. This assumption is conservative specifically for the debounce assessment, but it does not constitute a fully coupled energy balance or an experimentally validated restitution value for the assembled detector. Quantification of the actual effective restitution would require either instrumented impact testing or a coupled dynamic–elastoplastic contact model.

Moreover, a further limitation arises from the lumped-parameter representation of the pedal–spring subsystem. The dynamic model assumes that the pedal behaves as a rigid body at the assembly scale and therefore resolves the gross displacement, rebound, and settling response of the mechanism. It does not resolve the propagation and reflection of high-frequency elastic or plastic stress waves within the pedal, nor the very short-duration local micro-separation that such waves may produce at the wheel–pedal interface. Consequently, the predicted settling time characterizes the macroscopic rebound relevant to repeated magnet–reed crossings, rather than the complete high-frequency contact response. Resolving acoustic-wave-induced contact chatter would require an explicit transient continuum model with sufficiently fine spatial and temporal resolution. The present lumped model is therefore used only to evaluate the gross structural response relative to the firmware validation window.

On the other hand, wear and fatigue were not included in the plastic deformation analysis presented in this paper, because they represent degradation mechanisms that are distinct from residual plastic indentation. Wear would require a tribological formulation involving sliding distance, frictional conditions, wear coefficient, surface roughness, and environmental contamination, whereas fatigue would require a cyclic damage framework together with material fatigue data under the relevant contact state. Accordingly, the scope of the present analysis was intentionally restricted to evaluating whether repeated normal elastoplastic accommodation could compromise the vertical triggering margin of the reed-switch mechanism. In this sense, the proposed pedal should be interpreted as a replaceable and potentially sacrificial component within a short-term or mobile monitoring-oriented deployment concept, rather than as a permanent high-cycle contact element for certified railway infrastructure. Long-term wear, surface damage, and fatigue-related degradation will be addressed in subsequent field stages by tracking residual pedal indentation, surface condition, and triggering repeatability after accumulated wheel passages. Within this scope, the cyclic finite-element analysis should be interpreted as a shakedown-type assessment within the adopted reduced-order isotropic-hardening formulation, rather than as a complete wear-life, fatigue-life, or long-term ratcheting prediction. Over the simulated cycles, the predicted residual vertical indentation approached 0.70 mm and remained below the 5 mm triggering allowance. However, this model-dependent value should not be interpreted as a verified long-term upper bound; extended cyclic testing or a mixed-hardening analysis would be required to exclude progressive deformation over prolonged service.

Lastly, the present study is limited by the fact that the field validation was conducted under a limited range of practical conditions. Although the prototype was tested in real railway operation, the validation program did not yet include systematic long-term evaluation under contaminated surfaces, varying ambient temperatures, wet conditions, or externally intensified vibration disturbances. The detector housing and surrounding geometry were designed to allow water drainage and to reduce standing water accumulation around the sensing region; however, extended validation under such environmental conditions remains necessary. These factors may influence the repeatability and practical robustness of the detector over extended service intervals. Furthermore, the mechanical model primarily considers the vertical wheel–pedal interaction and does not explicitly resolve lateral or multi-axial wheel dynamics. In real railway operation, wheelset hunting, flange-angle variation, local track irregularities, and suspension dynamics may modify the detailed contact kinematics at the wheel–pedal interface. Accordingly, broader environmental and operational validation, together with extended lateral contact sensitivity or multi-body dynamic analysis, remains an important direction for future work.

## 9 Conclusions

This study presented an open-source, low-power, pedal-based wheel detector intended for non-safety–critical railway monitoring applications. The proposed system combines a mechanically preloaded pedal, a reed-switch-based triggering arrangement, and an ultra-low-power electronic subsystem to provide a simple and field-deployable wheel-passage monitoring node.

The results showed that the selected 24 mm preload is the minimum value required to suppress secondary penetration, thereby improving the dynamic stability of the pedal response. At 90 km/h, the adopted linearized dynamic model predicted full damping within 17.08 ms, remaining below the selected 30 ms digital filtering window. In addition, the damping sensitivity analysis indicated that this timing margin is preserved under reasonable variation of the assumed structural damping. Within the adopted reduced-order 2D plane-strain model with isotropic hardening, the cyclic elastoplastic analysis predicted a shakedown-like response, with residual vertical indentation approaching 0.70 mm and remaining below the 5 mm triggering allowance over the simulated cycles. This model-dependent value should not be interpreted as a verified long-term upper bound for accumulated deformation.

Field validation under real railway operating conditions was performed over the 0–90 km/h range using a single railway vehicle with known wheel passages on the instrumented rail. A total of 500 monitored wheel-passage events were evaluated, with no missed detection observed during the test program and no visible wheel-flange deformation identified after the tests. Within the investigated operating envelope, these results support the feasibility of the proposed concept for stable single-event generation and practical low-power field deployment.

The proposed detector is not intended to replace certified train-detection or axle-counting infrastructure, but rather to support non-safety–critical railway monitoring applications such as temporary wheel-passage logging, traffic-density estimation, train-frequency observation, auxiliary triggering of local data-acquisition systems, maintenance support measurements, and open-hardware field experimentation. In this sense, it should be interpreted as a low-cost, monitoring-oriented hardware platform and event-generation node for short-term deployments, portable monitoring applications, and distributed sensing studies in which wheel-passage events are used as inputs for higher-level infrastructure or operational monitoring workflows. Long-term environmental validation, extended multi-axial dynamic modelling, and accumulated wear and fatigue assessment remain important directions for future work.

## CRediT authorship contribution statement

**Eren Erdi:** Writing – review & editing, Writing – original draft, Validation, Software, Methodology, Conceptualization. **Emrah Sarioglu:** Visualization, Conceptualization. **Baris Oguz Gurses:** Supervision. **Aysun Baltaci:** Supervision.

## Funding

This research did not receive any specific grant from funding agencies in the public, commercial, or not-for-profit sectors.

## Ethics statements

The authors declare that this work complies with the journal ethics policies and did not involve human subjects or animal experiments.

## Declaration of competing interest

The authors declare that they have no known competing financial interests or personal relationships that could have appeared to influence the work reported in this paper.
